# Antioxidant Naturally Occurring Pleiotropically Acting Bioactive Compounds, as Polymeric Nanotherapeutics Against Autoimmune Diseases Progression

**DOI:** 10.3390/cimb47060411

**Published:** 2025-06-01

**Authors:** Panagiotis Theodosis-Nobelos, Fani-Niki Varra, Michail Varras, Georgios Papagiouvannis, Eleni A. Rekka

**Affiliations:** 1 Department of Pharmacy, School of Health Sciences, Frederick University, Nicosia 1036, Cyprus; 2 Medical School, Democritus University of Thrace, 68100 Alexandroupolis, Greece; 3 Fourth Department of Obstetrics and Gynecology, ‘Elena Venizelou’ General Hospital, 11521 Athens, Greece; 4 Department of Pharmaceutical Chemistry, School of Pharmacy, Aristotle University of Thessaloniki, 54124 Thessaloniki, Greece

**Keywords:** autoimmune disease, oxidative stress, antioxidant bioactive compounds, inflammation, targeted drug delivery, pharmacokinetic improvement, controlled drug release

## Abstract

Autoimmune diseases are driven by chronic inflammation and oxidative stress, thus requiring innovative therapeutic approaches. Polymeric nanotherapeutics incorporating antioxidant bioactive compounds offer a promising strategy for immune modulation and enhanced drug delivery. This review explores the application of polymer-based nanocarriers for improving the solubility, bioavailability, and targeted delivery of antioxidant compounds in autoimmune disease treatment. A comprehensive analysis of recent advancements in polymeric nanoformulations, including poly(lactic-co-glycolic acid) (PLGA), polyethylene glycol (PEG), chitosan, and hyaluronic acid, was conducted. The therapeutic efficacy of various antioxidant-loaded nanoparticles has been assessed in both preclinical and clinical studies. Phenolic antioxidants, such as resveratrol, curcumin, quercetin, and epigallocatechin-3-gallate, exhibit potent anti-inflammatory effects; however, their poor solubility limits their clinical application. Nanocarriers such as dendrosomes, tannic acid-based reactive oxygen species (ROS)-scavenging nanoparticles, and folic acid-functionalized systems enhance drug stability, controlled drug release, and macrophage targeting. Carotenoid and bilirubin nanoparticles further demonstrate immunomodulatory effects in multiple sclerosis, psoriasis, rheumatoid arthritis, and inflammatory bowel disease. Polymeric antioxidant nanotherapeutics provide targeted and sustained drug delivery, offering improved efficacy and reduced toxicity. Future research should focus on optimizing these nanocarriers for clinical translation and patient-centered therapeutic strategies.

## 1. Introduction

Autoimmune diseases are a heterogeneous group of chronic disorders in which immune cells (B or T lymphocytes) mistakenly attack the host’s own cells and tissues, leading to persistent inflammation and tissue damage. These diseases, including rheumatoid arthritis (RA), multiple sclerosis (MS), inflammatory bowel disease (IBD), psoriasis, and type 1 diabetes (T1D), affect millions of people worldwide and pose significant healthcare challenges. Antibody production can be used as a promising biomarker for the diagnosis and classification of the disease [1,2]. Conventional therapies, such as nonsteroidal anti-inflammatory drugs (NSAIDs), corticosteroids, and immunosuppressants, provide symptomatic relief but are often associated with adverse effects, limited efficacy, and the risk of systemic immunosuppression. Thus, there is an urgent need for more effective and targeted therapeutic strategies with improved safety profiles [3,4].

One promising approach is the use of naturally occurring bioactive compounds with strong antioxidant and anti-inflammatory properties [5]. These compounds, such as polyphenols (resveratrol, curcumin, quercetin, and epigallocatechin gallate), carotenoids (lycopene, fucoxanthin), and other plant-derived molecules (tannic acid, folic acid, bilirubin, triptolide), exhibit various actions, including free radical scavenging, inhibition of pro-inflammatory cytokines, modulation of immune cell responses, and enhancement of cellular defense mechanisms [6,7]. However, despite their therapeutic potential, the clinical application of these compounds is hindered mainly by pharmacokinetic challenges such as poor solubility, low bioavailability, rapid metabolism, and limited target specificity [8].

These limitations can be overcome by polymeric nanotherapeutics, which have emerged as a novel drug delivery approach [9]. Nanocarriers, including polymeric nanoparticles, liposomes, dendrimers, and micelles, offer controlled and targeted delivery, enhanced solubility, prolonged circulation time, and site-specific accumulation of bioactive molecules [10,11]. Functionalized polymeric nanoparticles can be designed to respond to specific physiological stimuli, such as pH changes or oxidative stress, ensuring precise drug release in the inflamed tissues [12]. Additionally, polymer-based formulations can improve the stability of bioactive compounds, reduce systemic toxicity, and optimize their pharmacokinetic and pharmacodynamic properties [13].

Several studies have demonstrated the efficacy of polymeric nanocarriers for antioxidant-based therapies in autoimmune diseases. For instance, resveratrol-loaded ruthenium-based nanocarriers have shown improved macrophage targeting and controlled drug release upon external irradiation [14]. Similarly, curcumin-loaded polymeric nanoparticles have exhibited enhanced bioavailability and significant therapeutic effects in experimental autoimmune encephalomyelitis, ulcerative colitis, and psoriasis models [15]. Quercetin and naringenin, encapsulated in polymeric carriers, have also demonstrated potent anti-inflammatory effects in RA and other inflammatory conditions [16]. Moreover, innovative approaches, such as folic acid-functionalized nanoparticles for macrophage targeting and bilirubin-based nanotherapeutics for immunosuppression, have further expanded the scope of antioxidant polymeric nanomedicine [17]. Apart from modulation of inflammatory factors and immune response, polymeric and non-polymeric nanoparticles could be applied as advanced diagnostic and imaging tools, revolutionizing not only the prevention or therapy of diseases, but also their detection [18].

In this review, we provide a comprehensive overview of the latest advances in polymeric nanotherapeutics for antioxidant-based treatment of autoimmune diseases, with multi-targeting compounds, combining oxidative stress and inflammation modulation, together with several pharmacological characteristics such as antiapoptotic and cell protective, and improved pharmacokinetic behavior with their nanoformulations. We discuss the molecular mechanisms of bioactive antioxidants, their therapeutic applications, and the advantages of nanotechnology in the enhancement of their pharmacological potential, at the in vitro, in cellulo, in vivo, and clinical levels. By integrating nanomedicine with natural bioactive compounds, these emerging strategies hold promise for the development of safer and more effective multi-functional treatments for autoimmune disorders, ultimately improving patient outcomes and quality of life. For this review article, an electronic literature search was conducted in the PubMed and Scopus databases, emphasizing the research and review studies from 2010, relating to nanoformulations and polymeric formulations with natural antioxidant compounds against autoimmune diseases.

## 2. Phenolic Antioxidants

Resveratrol (RES) is a trihydroxy-stilbenoid natural phenol with reducing activity as well as macrophage transformation into M2 type, inhibiting the anti-inflammatory response and the secretion of pro-inflammatory cytokines via the M1 type macrophages [19]. Resveratrol modulates intracellular signaling by activating SIRT1 (sirtuin 1) and AMPK (AMP-activated protein kinase) pathways, enhancing mitochondrial biogenesis and antioxidant defenses. It inhibits NF-κB (nuclear factor kappa B), reducing inflammation, and suppresses mTOR (mammalian target of rapamycin) signaling, mediating autophagy [20,21,22]. These actions may contribute to its cellular protective and anti-aging effects [23]. However, the low water dissolution ratio and targeting of the macrophages may limit its application. Thus, a multifunctional Ruthenium-based nanocarrier was constructed in order to control precisely the release of RES, after irradiation of the nanocomposite (NC) [24]. For this NC, quadrilateral ruthenium (QRu) nanoparticles, able to induce photothermal effect, and the thermosensitive poly (lactic-co-glycolic) acid (PLGA) with dextran sulfate (DS) (for effective binding to the scavenging receptor of macrophage) were assembled. It resulted in significant improvement in water solubility, targeting of resveratrol, and reversal of the proportion between M1 and M2 cell types in vitro. In vivo accumulation at the lesion area, after the exogenous irradiation was observed, and the release speed was analogous to the temperature, whilst at room or body temperature, the NC was stable. Furthermore, the uptake from macrophages in cellulo was energy dependent, with endocytosis being accomplished at increased temperature, without being able to enter in HUVECs cells, indicating the potential of the NC to be scavenged by specific binding with scavenger receptors of the immune cells, with inducible NO synthase and CD80 protein levels being statistically significantly lower in the treated cells, and Argimase-1 and CD20 increase respectively. M1-induced inflammatory response inhibition was followed by an increase in the inflammatory cytokines TNF-α (tumor necrosis factor α), IL-1β (interleukin 1β), and IL-6, and a decrease in the anti-inflammatory cytokines IL-4 and IL-10, especially after laser application (photothermal effect) together with NPs administration. These results were also verified in vivo with cytokine levels and histological improvement in mice; however, no clinical results have verified this interesting hypothesis of these resveratrol-loaded nanoparticles. Furthermore, the QRu provided the ability for photoacoustic imaging, with analysis of the distribution and imaging of the complex. The synthesis took place by mixing the RuCl_3_ with PVP (vinylalcohol polymer), and their collection was made by centrifugation. The resulting mixture was sonicated with PLGA and RES. After the addition of PVP and the evaporation of the solvent, the QRu-PLGA-RES nanoparticles (NPs) were collected by respective centrifugation. The final QRu-PLGA-RES-DS resulted from the conjugation of dextran sulfate [with the addition of 1-Ethyl-3-(3-dimethylaminopropyl)carbodiimide HCl (EDC-HCl) and N-Hydroxysulfosuccinimide (NHS)].

Curcumin (Cur) is another highly antioxidant and anti-inflammatory natural compound with low solubility in water, with decreased absorption and rapid elimination, yielding low plasma and tissue concentration. Lipid-based nanosystems [nanolipid carriers (NLCs), solid lipid nanoparticles (SLNs), and liposomes] and polymer-based nanosystems are experimentally used for MS manipulation [25]. Additionally, the application of NPs of Cur has pointed out remarkable results as a dendrosome nanostructure. This was comprised of oleic acid and polyethylene glycol (PEG) of 2000 Dalton molecular weight, which were esterified by oleoyl chloride and methoxyPEG with triethylamine [26]. Curcumin was embodied via mixing the solution of Cur in acetone with the nanocarrier solution. The effect of this polymerized form of nano-curcumin (PNC) has been tested in Lewis rats’ autoimmune encephalomyelitis, induced by complete Freund’s adjuvant, with inactivated Mycobacterium tuberculosis for amplification of immunization [27]. PNC was administered intraperitoneally daily, either on the first day of the immunization, for testing of its prophylactic effect, or from the twelfth day post-immunization. The PNC pretreatment succeeded in significantly lower peak and cumulative values of EAE (experimental autoimmune encephalomyelitis) compared to the control group (*p* < 0.05 and *p* < 0.0001, respectively), and the curcumin alone treated group (*p* < 0.001), with higher scores of myelination (especially in the pretreated group), as well as BBB (Blood Brain Barrier) integrity, possibly derived partly from the correction of the balance of pro- towards the anti-inflammatory genes expression and the improved oxidative stress markers like heme oxygenase-1 (HO-1), nuclear factor erythroid 2-related factor 2 (NRF2) and the inducible NO synthase (iNOS). These promising therapeutic effects of Cur were also recorded in MS patients after oral administration of Sina-Curcumin, a nano-curcumin product, with encapsulated curcumin in nano-micelle [28]. The mRNA expression and release of inflammatory mediators [IL-6, IL-1β, IFN-γ (interferon γ), TNF-α, CCL2 (C-C motif ligand 2) and CXCL8 (CXC motif ligand 8)] was significantly decreased, whether the expression of sirtuin-1 and scurfin (Foxp3) was increased, mitigating the elevated expression of pro-inflammatory cytokines that promote MS pathogenesis. These results verify also the in vitro findings of curcumin nanotherapeutics for MS manipulation, although differences in the nanoformulations may limit the ability to extract solid conclusions for the potential of nanocurcumin derivatives, since although experimental autoimmune encephalomyelitis is a well applied in vivo model for MS replication, it is not able to fully convey the circumstances that evolve during the MS progression.

The curcumin nanotherapeutics release and activity have also been tested in the autoimmune ulcerative colitis (UC) and inflammatory bowel disease (IBD), since its oral efficiency is degraded by the low mucus penetrating potency and the uncontrolled drug release. In ulcerative colitis, PLGA nanoparticles were used in combination with a nonionic surfactant polyol, the pluronic F127 (for facilitation of the mucus penetration and the uptake by macrophages), and these nanoparticles were loaded with catalase and curcumin [29]. Catalase was used for the transformation of hydrogen peroxide in the inflamed area into oxygen, which increases the release rate of the NPs. The NPS were prepared by a double emulsion solvent evaporation method, by dissolving bovine serum albumin and PLGA, PF127, and Cur in dichloromethane. The final aqueous and oily phases were combined, sonicated, and then inserted into a PVA water solution to form the final water-in-oil-in-water emulsion. The resulted NPs had narrow distribution of size and negative zeta potential (maintaining their well-defined spherical structure and smooth surface morphology even after incubation in the H_2_O_2_), mucus-penetrating and cell internalization capacity, and were able to narrow down the secretion of the inflammatory cytokines (TNF-α, Il-6, IL-12) and increase the anti-inflammatory IL-10, compared to the non PF127 or catalase containing NPs in vitro. Additionally, PLGA-CUR-NPs showed high antioxidant activity in LPS (lipopolysaccharide) induced Raw 264.7 macrophages, with the intrinsic antioxidant activity and the catalase incorporation being involved in the potency of the NPs. NPs could also efficiently be released from the hydrogel and penetrate the colonic mucosa in vivo, which may be implicated in the in vivo therapeutic outcomes in UC (low body weight loss and colonic shortening of the treatment group, together with cytokine modulation).

**Figure 1:**
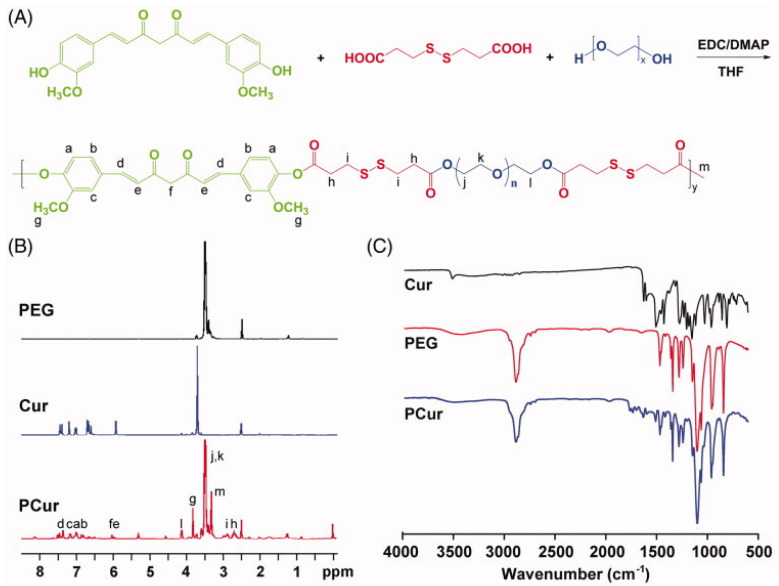
Synthetic scheme of PCur conjugate (**A**); representative ^1^H NMR (**B**) and FT-IR (**C**) spectra of original reactants (PEG and Cur) and the final product (PCur) [30]. Curcumin is covalently bonded with PEG via a 3,3-dithiodipropionic acid linkage, and the esterification took place with EDC and DMAP, with the resulting product incorporating the structures of Cur and PEG, as the ^1^H NMR and the FT-IR spectra depict.

Respectively, for the inflammatory bowel disease curcumin, covalently bonded with PEG, via the dicarboxylic compound 3,3-dithiodipropionic acid [the carboxylic groups were activated with EDC (1-Ethyl-3-(3-dimethylaminopropyl)carbodiimide) and the esterification took place in the presence of DMAP (4-dimethylaminopyridine) after stirring for 24 h], was used, trying to exploit the bacterial reductive ability for the hydrogenation of the disulfide bond [30] (Figure 1). The improved solubility, due to the hydrophilic PEG moiety [and in vitro stability at low pH (in the presence of hydrochloric acid), or in the presence of glutathione], and the relatively neutral surface potential lead to the passive diffusion to the compartments of increased blood circulation, as is the inflamed areas, with limited release at the pH of the gastrointestinal tract, offering improved bioavailability and enhanced targeted pharmacokinetic behavior, as the dextran sodium sulfate (DSS) induced murine model of IBD demonstrated (curcumin polymer was administered orally). Cur NPs in this study showed low toxicity in Caco-2 treated cells, with enhanced cellular permeability in cell lines and in vivo in Spraque-Dawley rats, and intact epitheliums and structure of the colon in vivo, accompanied by antioxidant capacity, as the lipid peroxidation products and antioxidant enzymatic activity showed, accentuating the role of the conjugate of Cur as a colon targeting candidate.

Another field of autoimmunity in which Cur nanoparticles have been tested is psoriasis [31]. In this extensive study, a RRR-α-tocopheryl succinate-grafted-ε-polylysine conjugate (VES-g-ε-PLL) (an amphiphilic ε-polylysine-based graft polymer) was used for the loading of curcumin, forming small (24.4 nm) cationic NPs, with positive Zeta potential (19.6 mV) and increased cell-penetration of the stratum corneum of the skin in vivo. For the improved attachment to the skin, silk fibroin (SF), a natural biological adhesive, was used, with adjustable biodegradability to non-toxic products, and biocompatibility [32]. The initial Cur-NPs were prepared via dynamic dialysis and then mixed with the SF solution (under sonication) to yield the resulting gel. The VES-g-ε-PLL NPs could effectively encapsulate Cur up to almost 80%, whilst the SF incorporation offered a prolonged release, but also a high skin permeability profile. Additionally, the in vivo effects on miquimod-derived psoriasis were significant, since the Clinical Psoriasis Area and Severity Index (PASI) scores were decreased, from 4 to 1, for Cur NPs gel, and the histopathological features like hyperplasia and leukocytic infiltration were inhibited, whilst the score was 2 for the non-NP curcumin. This result is also reflected in the TNF-α positive cells that were remarkably decreased (2.2 fold compared to Cur-gel) in the NPs-gel group. Similar inhibitory effects were also seen at the levels of NF-κB and IL-6. Another promising technique for psoriasis is described by Zhang et al. [33]. It refers to covalently linking hyaluronic acid with propylene glycol-based ethosomes, as a skin drug delivery system, enhancing the transdermal retention in psoriasic skin in vivo, 2.3 and 4 times respectively than ethosomes and propylene glycol solution, whilst the inflammation of the skin led to improved drug accumulation at the area, with alleviation of the inflammation, as shown by the reduction in the inflammatory markers mRNA levels [TNF-α, IL-17A, IL-22, IL-1β (tumor necrosis factor-α, interleukin-17A, -22 and 1β) and CCR6 (C-C chemokine receptor type 6, effector and regulator of T cell function)].

Furthermore, the oral effect of Cur nanoparticles as an adjuvant therapy for psoriasis, combined with acitretin (an alternative treatment for resistant psoriasis), has been tested [34]. For the selection of the NP system, diverse binary systems of Cur and hydrophilic polymers in various ratios have been produced with co-grinding at high energy vibration (24 Hz). The nanocrystals were investigated in terms of solubility, stability, and residual Cur content. PVP30 was chosen as a stabilizer, and the final stabilized nanoparticles were used for the double-blind, randomized, placebo-controlled phase III trial for moderate to severe psoriasis, in co-treatment with acitretin, with superior reduction of the PASI scores compared to acitretin alone. Furthermore, Cur NPs seem to have enhanced permeability compared to Cur, as Papp values were up to 12.3 times higher (evaluated with a parallel artificial membrane permeability assay to predict the passive intestinal absorption). Additionally, as far as the safety profile of the co-administered compounds is concerned, the acitretin-treated group appeared to have a significant elevation of the total cholesterol levels compared to the Cur-NPs co-treated, whilst LDL and HDL cholesterol levels were similar in both groups. As for other toxicity issues, only one patient reported nausea and vomiting, and some others experienced mild cheilitis on the arm and peeling of palms (8 patients in total).

Quercetin (Quer) is a flavonol with a wide range of biological properties and a quite safe toxicological profile, but with low water solubility and a high total polar surface area of 127 Å^2^ that leads to its poor gastrointestinal and epidermal absorption and its rapid excretion [35,36]. Various methods for confronting the physicochemical defects of Quer have been applied for the treatment of RA, like nanoformulations via spontaneous emulsification techniques or glycolic acid-capped cadmium telluride quantum dots with promising results [36,37]. In the first case [36], Quer nanoemulsion was non-toxic (in HIG-82 cells) and had an increased anti-inflammatory profile on lipopolysaccharide-induced TNF-α production (in RAW 264.7 cells). It presented improved membrane permeation ability and rheological properties, and prolonged paw oedema inhibitory potency in rats, with a good texture and non-irritating profile. In the second case [37], the oral administration of quantum dots nano-carrier showed high antioxidant and radical scavenging activity in four in vitro experiments, as well as in the adjuvant induced arthritis in Wistar rats, where decreased lipid peroxidation and statistically significant increase in the tested antioxidant enzymes [SOD, glutathione peroxidase (GPx), catalase] was observed. These effects were accompanied by a significant reduction in inflammatory markers, as C-reactive protein, rheumatoid factor, red and white blood cell count, and erythrocyte sedimentation rate (ESR), with parallel cartilage regeneration. Another oral antiarthritic polymeric application of Quer was presented by Souza et al. [38] with the application of the naturally occurring pectin and casein for the encapsulation of Quer and the oral administration in arthritic (adjuvant-induced) rats, at a dose of 10 mg/kg. There was an efficient encapsulation, as the x-ray diffractogram indicated, with oxidation normalization, as the TBARS (thiobarbituric acid reactive substances formed as a byproduct of lipid peroxidation), GSH (glutathione), and protein carbonyl groups levels in the rats’ liver and brain dictate. However, low clinical improvement in the paw volume was observed, accentuating the low anti-inflammatory potency in this procedure. Apart from oral administration, epidermal application of Quer could be a tool for skin autoimmunity disorders. Hyaluronic acid (HA) covalent bonded conjugates with Quer and other important catechins have been synthesized with epichlorohydrin (EPH) as an intermediate linker [39]. EPH was mixed with NaOH aqueous solution of catechin, and the resulting intermediate was added to the HA aqueous solution to give the polyphenol-grafted hyaluronic acid. The adhesion of the conjugates on polypropylene surfaces was more than 10-fold increased compared to HA alone, with slightly higher thermal stability than the neat HA macromolecule. The antioxidant potency, measured by the in vitro interaction with the DPPH radical, was strong and completely related to the flavonoid concentration and the adhesion strength of the conjugates (relative to the number of hydroxyl and catechol groups in the formulation), whilst HA showed no antioxidant activity. In this antioxidant direction, the ionic gelation properties of the polymeric structure also resulted, with the strong coordination between FeCl_3_ and the catecholic hydroxyl groups of Quer, showing the metal chelatory antioxidant activity as well as the self-healing behavior of the resulting gel.

Similarly to Quer, naringenin (NAR), a flavanone aglycone of naringin, possesses anti-inflammatory and antioxidant properties, but unfavorable physicochemical characteristics [40]. For these reasons, NAR liposomes with polymeric content have been formulated via the combination of NAR-containing lecithin and chitosan solutions, with the resulting mixture being centrifuged and re-dispersed several times to remove the surfactant excess, with final lyophilization at the end of the procedure [41]. This binary mixture showed positive (+32 mV) zeta potential with 190 nm size and substantial (85%) entrapment efficiency. The NAR-loaded lipid NPs (orally, 40 mg/kg for 28 days) decreased the CFA (complete Freund’s adjuvant) induced rat (male albino) ankle swelling and the inflammatory markers, TNF-α, IL-6 and cyclo-oxygenase-2, significantly (*p* < 0.001), whilst the histopathological changes in the joint were less pronounced and with diminished number of inflammatory cells, than any other kind of liposomal NAR tested, whilst NAR administration was inferior to all the liposomal NAR-nanocarriers tested. Furthermore, the hematological values were also improved, especially by the LC-lipid polymer hybrid, compared to the other treatments, concerning the kidney, liver, red cells, platelets, and immune cells indicators. Another study also describes the usage of biodegradable, sustained-release NAR-PLGA NPs, produced by solvent emulsification and evaporation technique, with optimization with poloxamer-188, sodium deoxycholate, and PVA as stabilizers [42]. The final NPs had low particle size (<180 nm) and negative potential for sustained release efficiency, high entrapment potency (74%), and profound stability (no significant change in their morphology after 6 months storage at 40 °C and 75% relative humidity. Their release was also tested ex vivo and showed prolonged delivery for 24 h. The in vivo results on the chronic arthritic rat model yielded more than 2.5 units at the arthritic score, with an almost 22% reduction in the paw volume, and these reductions were greater than those achieved with NAR alone. Additionally, NAR NPs reduced serum C-reactive protein and rheumatoid factor levels in a manner similar to indomethacin, a very active classical NSAID. A similar pattern was observed for serum L-6, IL-10, TNF-α, and INF-γ (interferon-γ) levels. These results indicate the protective role of NAR liposomes and polymeric NPs for RA treatment, with the latter also offering increased sustained release.

Tannic acid (TA) is a polygalloyl form of tannin with a high antioxidant profile. TA has been combined with ROS scavenging nanoparticles for enhanced antioxidant effect and dose reduction, in order to reduce the harmful effects of rheumatoid arthritis [43]. In this report a polymeric phenylboronate (pPBA) was used as delivery system [produced by poly(maleic anhydride) and grafted PBA moiety], on a polymer backbone that would be able to form ester with catechol groups by simple mixing of pPBA (H_2_O_2_ consuming group) and TA (polyphenolic radical scavenger), with ROS responsive properties in the inflamed area. The best mixing ratio was 5:1 or 10:1, PBA to TA, since five outer catechols seem to react with PBA. However, the uniform distribution and the relatively small (250 nm) sample size made this proportion the chosen one. The uniform distribution was lost in the presence of hydrogen peroxide, accentuating the ROS scavenging potential of the polymer and the change of its shape, as tested by the horseradish peroxidase in vitro, in a dose-dependent manner. The anti-inflammatory effect of PTNG (phenylboronic acid-tannic acid nanogel) on murine macrophage (RAW264.7) cells was tested via stimulation with phorbol 12-myristate-13-acetate (PMA). PTNG decreased PMA-induced ROS production and TNF-α and IL-6 levels significantly. Additionally, the NPs were highly compatible and safe, since even at a concentration of 200 μM, viability over 80% was detected for RAW264.7 and colon cancer cell lines. The anti-inflammatory potential was also verified in vivo with peritonitis induction via zymosan, since neutrophils and myeloperoxidase activity reduction were observed, as well as preservation of macrophage levels by reduced movement to the lymph nodes [44]. Additionally, the immunosuppressive effect of TA NPs was tested on autoimmune type 1 diabetes (T1D) mice [45]. T1D was induced by streptozotocin, and C57BL/6 islets encapsulated with a multilayer coating [consisted of poly(N-vinylpyrrolidone) (PVPON), binded via hydrogen bonds with TA (hydrogen bonds were made between the carbonyl groups of PVPON and the phenolic groups of TA)] resulted in reduced immune mediated rejection and ROS scavenging, increasing significantly the survival time and quantity of the graft, and almost tripling the euglycemic state time. These effects were also derived from the immunomodulatory potential of the PVPON/TA, since decreased immune cell infiltration, inflammatory chemokines, and CD* T-cell effector responses were detected with an increase in the anti-inflammatory M2 macrophages. Furthermore, the intraperitoneal glucose tolerance test showed normal blood glucose levels in vivo with no statistical difference from the non-diabetic mice, although the clinical significance of these results remains to be elucidated, since the streptozotocin-induced T1D has a big differentiation from the human disease, despite the resemblances they may share.

(−)-Epigallocatechin-3-gallate (EGCG) is a major, mainly green tea-derived, antioxidant with anti-inflammatory and ROS, RNS scavenging characteristics [46]. In order to exploit the synergistic anti-arthritic activity of EGCG and that of glucosamine (GA), Zheng and his colleagues [47] prepared casein NPs encapsulated with EGCG and GA. The resulting EGC-NPs were synthesized by dissolution of both the compounds in water and NaHCO_3_ aqueous solution; both were added into a sodium caseinate solution in ratios 1:2:4 (*w*/*w*/*w*) for EGCG, GA, and casein, respectively, with the NPs being prepared with ultrasonication. The mean size of NPs was 186 nm, with entrapment efficiency reaching up to 86.8%. Their stability was high, preserving their physicochemical characteristics for a year after freeze-drying, whilst casein degradation in gastric fluids needed threefold more time to occur. NPs’ relative inhibitory activity on human fibroblast-like synoviocytes-rheumatoid arthritis cells was 20.8% more profound than the EGCG-GA mixture, a result that is consistent with the in vivo activity of the EGC-NPs in collagen-induced arthritis. The decrease in body weight was significantly less than that of the control or the celecoxib-treated group. The arthritic scores were significantly different (*p* < 0.01) from the control throughout the experiment, being similar to those of celecoxib and higher than those of the EGCG-GA mixture. Furthermore, the radiographic evaluation showed no bone erosion in the treated groups that was present in the control group. Thus, the decreased synovial hyperplasia, inflammatory cell infiltration, and connective tissue in the treatment group are logical and consistent with the radiographic results and the significantly lower inflammatory marker values. EGCG NPs have also been used in psoriasis alleviation [48]. A chitosan-based polymeric EGCG formulation was prepared by mixing chitosan and EGCG in the presence of pentasodium tripolyphosphate hexahydrate, followed by sonication of the resulting mixture [49]. The NPs induced differentiation of keratinocytes and decreased the propagation of inflammatory responses after topical application and offered significant amelioration of skin thickness, erythema, infiltration of inflammatory cells (mast cells, neutrophils, macrophages, and CD4^+^ T cells) and angiogenesis in imiquimod induced murine psoriasic model, with modulation of inflammatory cytokines and chemokines [Il-1α, IL-1β, IL-4, IL-5, IL-6, IL-12, IL-13, and IFN-α (interferon α)] [48].

Another encapsulation anti-arthritic method has been applied to eugenol, a phenolic compound with pleiotropic activities, in addition to its antioxidant properties [50]. An aggressive type of RA in rats, induced by CFA, with type II bovine collagen, was chosen, and chitosan was added at the extraction of eugenol, in the presence of tripolyphosphate solution, to obtain the final nanoparticles through centrifugation and encapsulation. Significant reduction was observed at the serum levels of malondialdehyde and Fork head Box O3 (FOXO3) protein (transcription factor expressed by regulatory T-cells), with decreasing effects also at the expression of Monocyte Chemoattractant Protein (MCP1)-1/CCL2 and TGF-β (tumor growth factor β), offering promising findings for RA inhibition. In another study, this time concerning the acute IBD, rosmarinic acid (RSA), another di-catechol compound, was applied in polymeric PEG form, yielding water-dispersible nanoparticles, for the improvement of its intrinsic poor water solubility and bioavailability [51]. The synthesis took place via dissolution of mPEG2K-amine [methoxy-poly(ethylene glycols amine; MW 2000] in the presence of N,N-diisopropylethylamine, and RSA was added for the final self-assembly of the nanoparticles (bearing 63.5 nm diameter and high colloidal stability for two weeks and hydrogen peroxide scavenging potential in vitro and at cellular level). At a second step, dexamethasone (DEX) was loaded in the NPs with release in oxidative stressful conditions and localization at the inflamed colon with dose dependent mitigation of inflammation (after intravenous administration in vivo), decreasing the disease activity index scores, the stunting and the histological damage of the colon, lowering the pro-inflammatory cytokine levels, whilst DEX-NPs showed higher therapeutic efficacy due to synergistic effect.

## 3. Folic Acid NPs

Folic acid (Vitamin B9), the synthetic form of folate, acts as a co-enzyme in mono-carbon metabolic reactions of purine synthesis and in methylation reactions in general, as is the transformation of homocysteine to methionine [52,53]. Furthermore, it possesses antioxidant, anticancer, cardio- and neuronal-protective actions, increasing the antioxidant capacity and the neutralization of reactive species in plasma [54,55]. Folate can be applied as a targeting moiety for the folate receptor b (FRb), in auto-immune diseases, as RA, since FRb is present in activated macrophages, rendering the prospect of interaction with pathogenic cell types possible, with little potency for toxic interactions with white cells [56].

Folate (FO) has been used as a targeting moiety for the cellobiose-coated polypropylene sulphide nanomatrix (NM) system, taken up by macrophages [57]. FO was conjugated with superoxide dismutase (SOD), an acid-labile enzyme, and propylene sulphide, which has catalase properties, for the conversion of peroxides to final non-active products. The synthesis of the final nanomatrix is quite interesting, starting with the propylene sulphide nanomatrix preparation using Pluronic F-127 and propylene sulphide [with 1,3-propane dithiol and diaza [5.4.0] bicycloundec-7-ene (DBU), as initiators], with termination of the reaction by the addition of oxygen and subsequent lyophilization of the polymer [58]. The polymer was mixed with a cellobiose solution under rigorous stirring, then added to water containing the utensil, giving the cellobiose-coated nanomatrix. This was afterwards lyophilized and washed with water for the removal of the loosely bound sugars. The folate conjugated SOD was prepared by covalent attachment of lysine residues of SOD with the carboxyl groups of folic acid, by the addition of 1-ethyl-3-(3-dimethylaminopropyl)carbodiimide. In the solution of FO-SOD, cellobiose-coated nanomatrix was added, giving the SOD-adsorbed matrix cores. The propylene sulphide polymer gave hydrogen peroxide EPH degradation ability. The FO-SOD cellobiose NM (FSODCE) had an oval shape with uniform coating, as the scanning electron microscopy dictates, with 104.5 nm mean particle size for the polymer core and 226.2 nm for the whole matrix. FSODCE exhibited an increase in antioxidant activity, tested by the nitro blue tetrazolium (NBT) assay method, compared to FO-SOD alone, with an increase in activity over time, indicating that the controlled release of the adsorbed enzyme may be the reason for this. However, these results are referred to as in vitro and in cellulo findings and are still in preliminary stage.

Folate covalently conjugated magnetic fibrin nanoparticles (MFNPs) have also been used as a contrast agent for the imaging of the RA lesions with MRI, since folate receptor-expressing activated macrophages are recruited at the knee joints of rats in antigen-induced arthritis [59]. MRI provides three-dimensional imaging of the soft tissues and the rheumatoid synovium [60]. For MFNPs, goat blood was used for the isolation of fibrin (by treatment of the blood with sodium acetate and hydrogen peroxide) and iron oxide (by red blood cells centrifugation and incineration), as a contrast agent. Iron hydrochloric acid solution and fibrin sodium hydroxide solution were combined to give MFNPs, whilst FO was conjugated by intermediate activation with EDC/NHS, and the final FO-MFNPs were purified via dialysis. The resulting NPs were spherical with a low size of 18.3 nm and with good internalization ability in macrophage cells in vitro and in vivo, with enhanced magnetic resonance imaging of the joints. As far as the imaging of RA is concerned, Pan et Chen produced another folic acid conjugated diblock polymer with polyethylene glycol–tert-butyl polyacrylate (PEG-b-PAA) and ultrasmall paramagnetic iron oxide (USPIO) [61]. The resulting FO-PEG-b-PAA/USPIO enhanced the quality of T2-weighted images for more than 24 h and offered early monitoring of lesions and synovial perfusion as well as the treatment response. As for the toxicity of the NPs, substantial cell viability (>82%, in RAW264.7 cell suspension) was documented, even after 48 h of co-incubation time, whilst the in vivo (from the venous blood of Sprague–Dawley rats) alanine aminotransferase (ALT), aspartate aminotransferase (AST), alkaline phosphatase (ALP), and blood urea nitrogen (BUN) levels remained the same with the control non-tested group, showing the safe profile of the NPs.

## 4. Carotenoids

Lycopene (Lyc) belongs to the family of carotenoids and possesses antioxidant properties due to increased resonance effect; however, its bioavailability is compromised due to low absorption (10–30%) and metabolic reactions, such as isomerization and oxidation, that occur [62]. Due to these pharmacokinetic and activity issues, Lyc water-in-oil nanoemulsion was formed by Moia and his colleagues, using Tween 80 and sonication [63]. The nanoformulation (NF-Lyc) was tested in male C57BL/6 mice in a model of RA induced by intra-articular injection of zymosan, where the NF-Lyc was administered i.p. (1 mg/kg), 1 day before the arthritis induction. The average diameter of the NF-Lyc was 241 nm with a polydispersity index of 0.284, uniformity, and an ability to aggregate, forming larger nanoparticles. NF-Lyc could inhibit the leucocyte, mononuclear and the neutrophil cells infiltration in the site of inflammation with higher efficacy (and dose dependent) of the nanodrug compared to the pure drug, a fact at least partly related to the EPR (Enhanced Permeability and Retention) effect of the NPs with parallel low oxidation of the incorporated Lyc, compared to the free Lyc. The nano-Lyc had low volume of distribution (0.404 L), ubiquitous tissue deposition and low excretion rate, accentuating the more pronounced preservation of the drug at the plasma and the inflamed areas, with low excretion by the kidneys and the liver (and negligible uptake in brain and ubiquitous distribution), as the 99 mTc radiolabelling of NF-Lyc showed. The increased inhibition of the MRP1, an efflux transporter protein (HEK293 T cells expressing MRP1 were used), accentuates the potency for interactions the NF-Lyc could have with other treatments, and the improvement on their absorption and their insertion into the cell that NF-Lyc could offer. In an analogous way, fucoxanthin, a marine carotenoid with anti-proliferative, antioxidant, and anti-inflammatory properties, has been loaded onto nanostructured lipid carriers (NLC) [64,65,66]. NLCs were prepared via the high shear homogenization method using bacuri butter and tacuma oil as lipids. NLCs were coated with chitosan with dimensions ranging from 250 to 400 nm, with chitosan increasing the size and improving the zeta potential, offering good bioadhesion, and increased uptake from psoriatic cells (using psoriatic-like cellular model), compared to the fibroblasts, where the cellular viability was enhanced (sign of reduced uptake), rendering chitosan coated NLCs loaded with fucoxanthin as an anti-psoriatic approach with skin integrity preservative effects. These NLCs also showed good in vitro biocompatibility (in fibroblast cells), with fucoxanthin loading providing an increase in NPs size, whilst the incorporation of the NLCs in pullulan film offered prompt interaction of the drug with the skin (according to in vitro bioadhesion testing).

## 5. Miscellaneous

The polymeric method in antioxidant compounds has also been applied to reach optimal geometric conditions of the antioxidants. This is the case with phosphatidylserine (PS). PS is shown to inhibit phosphatidylcholine hydroperoxides and the products of the thiobarbituric acid test on iron-induced lipid peroxidation [67]. In liposomes it has been able to mimic apoptotic cells function, reprogramming of macrophage formation, diminishing the release of inflammatory cytokines like IL-1β and increasing the anti-inflammatory ones (IL-10), affecting the T-cells and T-reg cells proportion [macrophages underwent a phenotypic change from interleukin (IL)-1β-producing to IL-10-producing cells after the phagocytosis in adjuvant arthritic rats, whilst the same potency was observed in LPS stimulated macrophages, with parallel inhibition of the rapid activation of p38 mitogen-activated protein kinases (MAPK), nuclear factor (NF)-κB and the receptor activator of NF-*κ*B, and activation of extracellular signal regulated kinase] [68]. This effect on innate immune cells is optimal when PS is incorporated into rod-shaped PLGA, whilst spherical liposomal PS or cylindrical PLGA particles offer less potency in MS and organ rejection in in vivo experiments [69]. The PS nanoparticles (80 × 320 nm) reduced myelin T-cell activation, decreasing the levels of interferon-γ (INFγ), tumor necrosis factor α (TNFα), and interleukin-6 (IL-6). The results were improved, compared to the liposomal and cylindrical particles, in a reversed to dose manner, reaching up to very statistically significant levels for 12.5 μM of PS, accentuating the role of biomaterials and of the geometric manipulation in the programming of cellular signaling in autoimmune conditions, paving the way also for organ rejection manipulation.

Embelin (2,5-dihydroxy-3-undecyl-1,4-benzoquinone) is a naturally occurring lipophilic para-benzoquinone derived from Embelia ribes plants, with antioxidant characteristics and 5-lipoxygenase and prostaglandin E2 synthase-1 inhibitory potency [70]. The effect of embelin-loaded chitosan NPs on the attenuation of experimentally induced RA, via subcutaneous injection of complete Freund’s adjuvant (CFA), showed significant and dose-dependent reduction in arthritic score and paw volume after the 15th day of the CFA administration (first day of NPs application by oral gavage), even better than naproxen, a well-established classical non-steroidal anti-inflammatory drug, at a dose of 50 mg/kg of embelin [71]. The synthesis of the NPs took place via an ionotropic gelation method (by the addition of sodium tripolyphosphate to the final mixture of chitosan and embelin and successive stirring and centrifugation of the resulting cross-linked chitosan-embelin NPs). Their in vivo effect on hematological markers of rats as the white blood cell count, hemoglobin and erythrocyte sediment rate were statistically significantly improved (*p* < 0.05) and the oxidative stress was reversed with up-regulation of the glutathione and superoxide dismutase (SOD) levels and down-regulation of malonyl-dialdehyde, the final lipid peroxidation product, nitrogen monoxide, TNF-α, IL-1β and IL-6 in the inflamed area (paw tissues).

**Figure 2:**
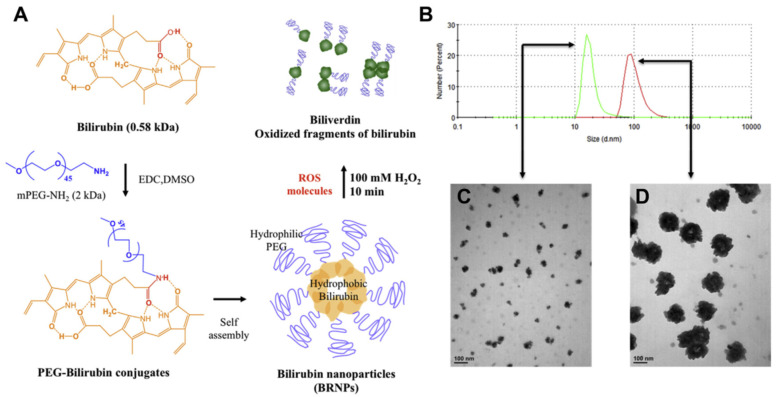
(**A**) Hydrophobic bilirubin molecules were conjugated with hydrophilic PEG molecules by EDC reactions. The PEG-bilirubin conjugates formed the bilirubin nanoparticles (BRNPs) by self-assembly in aqueous solution. These BRNPs scavenged free radicals and were converted into soluble biliverdin molecules or oxidized fragments of bilirubin, which have a dipyrrole structure. (**B**) The sizes of the BRNPs (40 mM) before or after treatment with hydrogen peroxide (H_2_O_2_) were determined by dynamic light scattering. Red: BRNPs. Green: BRNPs treated with hydrogen peroxide (100 mM for 10 min). (**C**) TEM images of degrading BRNPs after treatment with hydrogen peroxide. (**D**) TEM images of BRNPs without treatment with hydrogen peroxide. Scale bar ¼ 100 nm. (Reuse with permission from reference [75]).

A rather interesting, naturally occurring, heme metabolism compound with antioxidant and radical scavenging potential is bilirubin (BIL) [72]. However, its water insolubility makes it unfavorable for clinical practice. In order to minimize the insolubility and test the activity of BIL in the colon, and as an anti-inflammatory in general, Lee et al. covalently attached BIL with polyethylene glycol (PEG; molecular weight, 2000 gmol^−1^) amine, by amidation and formation of bilirubin NPs (BRNPs, after self-assembly with 94 nm size) [73]. BRNPs could scavenge ROS and minimize the concentration of hydrogen peroxide in vitro, and offer more than 80% cell viability (in CHO-K1 cells treated with hydrogen peroxide), even in concentrations 500 times less than that of hydrogen peroxide. These effects are accompanied by the excellent toxicological profile of the BIL-NPs that seem to cause no cell toxicity, in vitro or in vivo, in lung, liver, kidney, and spleen, or a change in the body weight in mice treated with them. In mice, intestinal inflammation was provoked by dextran sodium sulphate (DSS) oral administration for 5 days. On the fifth day, BRNPs were administered intravenously, using indocyanine green as a bioimaging compound. The NPs were significantly accumulated at the colon (as biodistribution imaging indicated), the site of inflammation, due to the increased blood circulation at the site and the leaky vasculature. The NPs-treated mice showed no weight loss and had substantial preservation of colon length compared to the non-treated group, whilst colonic myeloperoxidase activity was at the same level as the control (non-colitis-induced) group. The same group, recently, has also formulated hyaluronic acid (HA) BIL conjugated NPs [via reaction of bilirubin with ethylene diamine, using NHS (N-Hydroxysulfosuccinimide) and EDC (1-Ethyl-3-(3-dimethylaminopropyl)carbodiimide), and afterwards by binding with the carboxylic groups of HA] [74]. The resulted HA-BIL could self-assemble and accumulate at the inflamed colon diminishing the produced peroxyl radicals produced by 2,2′-azobis-2-methyl-propanimidamide, whilst it offered substantial (*p* < 0.01) protection of the H_2_O_2_ treated cells (HT-29 cells) and more significant (*p* < 0.001) stability of the resulted NPs compared to HA alone in the presence of hyaluronidase in vitro. The potential ROS scavenging effect and the hyaluronidase resistance offered protection of colonic epithelial cells, after DSS induced colitis (in C57BL/6 mice after oral delayed treatment), with preservation of the colon length and body weight and reduction in the colonic damage and the MPO activity (with parallel improvement of the inflammatory IL-1β and TNF-α, and of the anti-inflammatory ΙL-10 and tumor growth factor β markers). Another unexpectedly positive finding after the oral administration of the HA-BIL NPs was the modulation of the gut microbiome with an increase in richness and diversity, affecting the homeostasis of the intestinal barrier, with normalization of the expression of tight junction proteins. PEG-BIL NPs have also been studied for their ability to inhibit the pancreatic islet graft failure after transplantation [75]. The PEG size, the synthesis, and the self-assembly of the NPs are the same as those mentioned above by Lee et al. [73]. The bilirubin NPs after the radical scavenging are converted into biliverdin and other dipyrrole oxidized fragments, with a decrease in the size of their particles, due to the hydrophilicity of the resulting cores (Figure 2). The NPs could protect islet cells (extracted from male Sprague-Dawley rats) from oxidation (by decreasing ROS formation and lipid peroxidation [76]) and from the activated macrophages via inhibition in the cytokine release (TNF-α) at lower concentrations, whereas at higher (>50 μM) they did not offer protective effects and resulted in reduction in cell viability. These effects led to the xeno-transplanted islet prolongation of survival time by threefold, compared to BIL alone, with substantial inhibition of infiltration of the islets by inflammatory cells (in streptozotocin-induced diabetic mice transplanted with islets previously incubated with PEG-BIL NPs), as the levels of anti-CD45 antibody dictate. Additionally, a similar effect was observed for the nonfasting blood glucose levels increase in the transplanted mice, preserving the glucose levels low, for 10 days compared to the bilirubin-treated group (three times more than the BIL group), indicating the protection of the islet cells that the NPs offered.

Triptolide (TRP) is an epoxy diterpene lactone from Tripterygium wilfordii, an antioxidant with anti-inflammatory and anti-fibrotic effects, partly derived from its IKKβ and NF-κB inhibitory activity, leading to cytokines reduction [77]. TRP’s toxicity, also due to its multi-epoxy moieties and its low solubility, makes its application arduous. Ren and his colleagues tried to combine TRP and CCPA (2-chloro-N6-cyclopentyl adenosine), a selective A1 receptor agonist that plays a crucial role in analgesia, using acupoint nanocomposite gel (ANG) for RA pain and inflammation decrease [78]. TRP was incorporated with human serum albumin (HSA, via TRP-induced self-assembly of HSA, leading to TRP-HSA, in order to overcome the toxicity and poor solubility issues of triptolide, and the nanocomposite hydrogel derived by the self-assembly of a six-peptide protein was mixed with the TRP-HSA and CCPA, forming a homogenous, free-flowing hydrogel. The TRP-HAS NPs were spherical with uniform size, exhibiting drug loading and encapsulation efficiency of 6.19% and 68.21%, respectively, and with good stability. These spherical NPs could be efficiently loaded at the nanocomposite hydrogel with broad linear viscoelastic region and shear-thinning behavior, with no changes in the behavior of the hydrogel, accentuating its potential to act as a solid depot for the acupoint delivery of the ingredients, with sustained release of the CCPA and higher rate of release of the TRP at acidic conditions (due to change in the HAS conformation), a fact that complies perfectly with the reduced pH levels at the inflamed area of the arthritis joints. The resulting NPs, with acupoint injection, could target the inflamed joints in vivo, with lesser accumulation in the liver (and thus better pharmacokinetic and toxicological profile), compared to the non-acupoint injection, with the effect lingering even after 48 h. The area under the curve was for the nanocomposite hydrogel higher 20.2 and 3.6 times than for the free and the TRP-HSA alone. In CFA-induced RA, the nanocomposite gel could offer improvement, almost to the normal values, of the paw withdrawal latency and threshold and of the paw thickness, volume, and arthritis score. The decrease was comparable to that of the untreated group, reaching up to 75%, and all other approaches, including gel mono-treatment and non-acupoint usage, were less effective, although in many cases, this difference was non-statistically significant. Furthermore, ANG could improve the delay of the disease progression and alleviate the pain. Synovitis, progressive cartilage, and bone destruction were reversed to normal effects, and IL-1β, IL-6, and TNF-α levels were reduced in both cartilage and the synovium, with significant differences (*p* < 0.05) between the acupoint and non-acupoint strategy. Additionally, an increase in the anti-inflammatory IL-10 was recorded with a decrease in the ratio of the Th17 and the T regulatory cells (Tregs). These results were accompanied by the low toxicity observed by the histopathological analysis of major organs, including heart, liver, spleen, lungs, and kidneys, with acupoint ANG being more efficacious than the non-acupoint.

Apart from the antioxidant and anti-inflammatory compounds incorporated in polymers, there is another category of polymers that bear antioxidant and anti-inflammatory characteristics on their own, and these are the fucoidans, sulfated, fucose-rich polymers derived from microalgae, with ability to become bioavailable after oral administration, and with low toxicity problems, but with low half-life time, that may negatively affect the prolongation of its action [79,80]. Its anti-inflammatory characteristics derive from its selectin blockage capacity [81], inhibiting macrophage and CD4-positive T-cell infiltration [82], and the inhibition of the Complement [83] and enzymes such as matrix metalloproteases, hyaluronidases, and elastases, also offering antioxidant activity [80,84]. Thus, it can have positive results on osteoarthritis and neuronal protection, reversing the triggering of inflammation by LPS and the production of the highly reactive peroxynitrite [85]. Fucoidan microspheres have been used in techniques such as ionotropic gelation with cross linking agents and in coacervation forming poly-electrolyte complexes with other copolymers such as chitosan, hyaluronic acid, gelatin and casein, whilst it has been used as active molecule or as drug delivery system for the improvement of the pharmacokinetic properties of antioxidant and anti-inflammatory compounds such as curcumin and methotrexate [86].

## 6. Limitations and Concerns

Polymeric nanotherapeutics may offer several advantages over other nanotechnological systems for delivering antioxidant bioactive compounds. Their biodegradability, biocompatibility, and structural versatility allow for precise control over drug release profiles and surface functionalization. For example, unlike metallic or lipid-based nanoparticles, polymeric systems can encapsulate both hydrophilic and hydrophobic compounds, enhancing solubility and bioavailability. They may also, with proper modifications, offer lower toxicity risks and reduced immunogenicity. Additionally, polymers can be engineered to respond to specific stimuli (e.g., pH, enzymes), enabling targeted and sustained release at disease sites. These features make polymeric systems highly suitable for optimizing the therapeutic potential of naturally occurring antioxidant compounds. The choice of the nanotechnological drug delivery system is highly related to the molecular and physicochemical characteristics of the incorporated compounds and of the tissue that is going to be targeted, as well as to the pathophysiology of the disease for which it is intended. Additionally, the incorporation of tissue-targeting moieties in these nanostructures is also based on the specific antigens and cellular characteristics of the disease or condition, in order for targeted delivery to be successful.

Despite their promising potential, polymeric nanotherapeutics face several limitations that hinder their widespread clinical translation. Key concerns include safety and toxicity, particularly related to the long-term biodegradation and accumulation of polymeric carriers, which may induce immunogenicity or off-target effects. Manufacturing feasibility remains another critical barrier, as the reproducible large-scale production of nanocarriers with consistent physicochemical properties is technically challenging and costly. Regulatory hurdles are compounded by the lack of standardized protocols for evaluating toxicity and efficacy in preclinical models. Moreover, the complex interaction of nanocarriers with the biological milieu leads to unpredictable pharmacokinetics and biodistribution, limiting translational success. Existing research has largely focused on in vitro studies and small animal models, revealing a notable gap in robust, long-term in vivo assessments and clinical trial data. Future directions must prioritize the development of biodegradable, immuno-neutral polymers and nanoformulations, scalable fabrication methods, and real-time tracking systems for nanoparticle biodistribution. Additionally, regulatory frameworks should evolve to accommodate the unique characteristics of nanomedicines. Collaborative efforts between academia, industry, and regulatory bodies are essential to overcome obstacles in clinical translation, including patient heterogeneity, personalized dosing, and economic considerations. Bridging these gaps will be crucial for realizing the therapeutic promise of polymeric nanocarriers in the delivery of antioxidant bioactive compounds.

## 7. Conclusions and Future Perspectives

**Figure 3:**
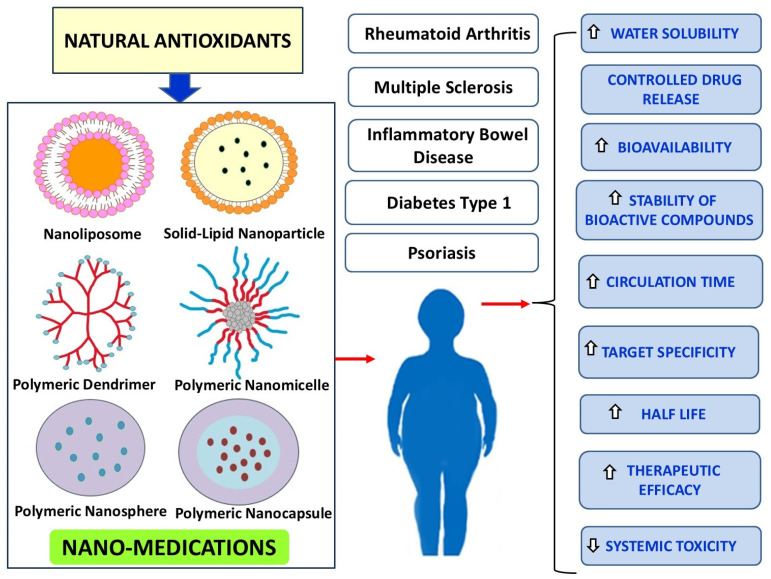
Nanostructures for natural antioxidants incorporation for immune regulation and physical properties improvement.

The incorporation of naturally occurring antioxidant bioactive compounds in polymeric nanotherapeutics offers a promising approach to mitigating autoimmune disease progression. Conventional antioxidants, such as resveratrol, curcumin, quercetin, naringenin, tannic acid, epigallocatechin gallate, and other polyphenols, exhibit potent anti-inflammatory and immunomodulatory effects. However, their therapeutic potential is often limited by poor solubility, rapid metabolism, and suboptimal bioavailability. To overcome these limitations, nanotechnology-based drug delivery systems, including polymeric nanoparticles, liposomes, micelles, and nanoemulsions, have been developed to enhance stability, bioavailability, and targeted delivery to inflamed tissues. Several studies highlighted in this manuscript demonstrate the efficacy of these nanosystems in experimental autoimmune disease models. Resveratrol-loaded quadrilateral ruthenium nanoparticles improved macrophage polarization, curcumin-dendrosome nanostructures enhanced myelination and blood-brain barrier integrity in multiple sclerosis, and quercetin nanoemulsions significantly suppressed inflammatory responses in rheumatoid arthritis. Similarly, folic acid-functionalized nanoparticles provided targeted imaging and therapy in arthritis models, while bilirubin-PEG nanoparticles exhibited radical scavenging activity in colitis and pancreatic islet transplantation. Moreover, innovative polymeric carriers such as fucoidans, phosphatidylserine-coated nanoparticles, and triptolide-human serum albumin formulations further optimize the immunomodulatory and anti-inflammatory effects of bioactive compounds. These nanocarriers facilitate controlled release and prolonged circulation, reducing systemic toxicity while enhancing therapeutic efficacy. The integration of these nanotherapeutics into clinical practice requires further studies to evaluate their long-term safety, pharmacokinetics, and large-scale manufacturing feasibility. Nevertheless, the advancements in nanotechnology-based delivery systems provide a promising avenue for improving the treatment outcomes of autoimmune diseases. By harnessing the pleiotropic effects of natural antioxidants and optimizing their pharmacokinetics through polymeric nanocarriers, the future of autoimmune disease therapy is poised for significant breakthroughs (Figure 3). Table 1 summarizes the physicochemical characteristics and the therapeutic efficacy of the free antioxidants, described in the text, and their nanocarrier-mediated antioxidant delivery systems.

## Figures and Tables

**Table 1:** Recent developments in nanocarrier-mediated antioxidant delivery systems against autoimmune diseases.

Antioxidants	Physicochemical Characteristics and Therapeutic Effects of Natural Antioxidants	Nano-Carriers	Methods Preparing Nano-Antioxidants	Physicochemical Characteristics and Outcomes of Nano-Antioxidants
Resveratrol (Res)	(a) Natural Res’s molecular weight: 228.25 g/mol [87]. (b) Poor water solubility (20–30 μg/mL) [88]; poor oral bioavailability (<1%) [89]; rapidly metabolized in liver and bowel [89]. (c) It activates SIRT1 and AMPK pathways, modulates intracellular signaling and enhances mitochondrial biogenesis and antioxidant defenses. It inhibits NF-κB and reduces inflammation. It suppresses mTOR pathway mediating autophagy [20,21,22].	Ruthenium based nano-liposomes [24].	Hybridation of quadrilateral ruthenium (QRu) nanoparticles with poly lactic-co-glycolic acid (PLGA) nanoparticles [24].	(a) Significant improvement in water solubility compared to free resveratrol alone [24]. (b) Modulation of macrophage polarization in vitro, reversing the proportion between M1 and M2 cell types and regulation of controlled resveratrol release at the lesion site of MS patients [24].
Curcumin (Cur)	(a) Natural Cur’s Molecular weight: 368.39 g/mol [90]. (b) Poor water solubility (0.0004 mg/mL at pH 7.3) [91]. (c) Poor oral bioavailability [92]; rapidly metabolized and excreted [93]. (d) It inhibits secretions of pro-inflammatory cytokine, such as IL-4, IL-6, IL-8 and TNF-α [94]. It increases anti-inflammatory cytokine production, such as IL-10 and soluble intercellular adhesion molecule 1 (sCAM-1). It serves as ROS scavenger and increases serum GSH and SOD levels [94].	Nanoliposomes [25]; nanolipid carriers (NLCs) [25]; solid lipid nanoparticles (SLNs) [25]; polymeric dendrimeres [26]; polymeric nano-micelles [27,28,29,30,31,32,33,34].	Polymerization technique using esterification of oleoyl chloride and methoxyPEG 2000 in the presence of triethylamine and acetone [26].	(a) Nano-Cur’s size 10–250 nm [32,95]. (b) Increased solubility [30,96]. (c) Increased bioavailability [30,97]; positive Zeta potential (+19.6 mV) [31]; permeability (and targeted pharmacokinetic behavior) up to 12.3 times higher than free Cur [30,34]; prolonged release [32]. (d) It improves OS markers, such as HO-1, NRF2 and iNOS in EAE [27]. It reduces mRNA expression and released pro-inflammatory cytokines such as IL-6, IL-1β, IFN-γ, TNF-α [28,33] and increases anti-inflammatory genes expression in MS patients [28]. It reduces secretion levels of pro-inflammatory cytokines such as TNF-α, IL-6 and IL-12 and increases the anti-inflammatory levels of IL-10 in UC patients [29]. It reduces pro-inflammatory cytokines TNF-α, IL-17A, IL-22 and IL-1β in psoriasis [33].
Quercetin (Quer)	(a) Natural Quer’s molecular weight (302.236 g/mol) [98]. (b) poor water solubility (1 μg/ml) [99,100]; poor gastrointestinal and epidermal absorption (solubility 5.5 μg/mL and 28.9 μg/mL, respectively) [40]; high total polar surface area of 127 Å^2^; rapid excretion [35,36]. (c) It serves as ROS scavenger and metal chelator and inhibits xanthine oxidase (XO) and nitric oxide synthase (NOS) [35].	Nanoemulsions [36]; quantum dots (QDs) [37]; biopolymers such as pectin and casein [38]; hyaluronic acid (HA)-based nanoparticles [39].	Nano-emulsion formulation using spontaneous emulsification [36]. Nano-formulation of thioglycolic acid-capped cadmium telluride quantum dots [37]. Hyaluronic acid (HA) covalent bonded conjugates [39].	(a) Better permeability and rheological properties than free Quer [36], and efficient encapsulation [38]. (b) It increases the anti-inflammatory effects on lipopolysaccharide induced TNF-α production in RAW 264.7 cells [36]. (c) It inhibits paw oedema formation in RA model [36]. (d) It reduces lipid peroxidation and protein carbonylation, and inflammatory markers, such as CRP, RF, WBC count and ESR and increases GSH, SOD, GPx and CAT in adjuvant induced arthritic Wistar rats [37,38]. (e) HA-Quer shows adhesive, antioxidant, gelation, and self-healing properties in skin autoimmunity disorders [39].
Naringenin (NAR)	(a) Natural NAR’s molecular weight: 580.5 g/mol [40]. (b) Poor water solubility (1 mg/mL at 40 °C). (c) Poor oral bioavailability [101]; rapidly metabolized [40]. (d) It possesses anti-inflammatory properties by inhibiting leucocyte recruitment and preventing macrophages action [102]. It activates Nfr2 resulting in enhanced anti-inflammatory response [102]. It inhibits the activation of NF-κB and the secretion of pro-inflammatory cytokines such as IL-33, TNF-α, IL-1β and IL-6 [102]. Also, it inhibits iNOS [102] and MAPK [102] and suppresses the TLR4 receptor [102]. It possesses antioxidant properties inhibiting ROS production [102], scavenging free radicals [102] and increasing the activity of SOD, CAT, GPx, GST and GSH [102].	Chitosan-covered liposomes [41]; NAR-PLGA NPs [42].	Nanoprecipitation technique [41]. Solvent emulsification and evaporation technique [42].	(a) Nano-NAR’s size < 180–190 nm [41,42]; (b) Increased entrapment efficiency and sustained release [41,42]; (c) Increased stability [42]; positive Zeta potential (+32 mV) [41]; increased entrapment efficiency (74–85%) [41,42]. (d) It decreases the CFA induced rat ankle swelling and the inflammatory markers TNF-α, IL-6 and COX-2 [41]. It reduces paw volume (−22%) on chronic arthritic rat model, CRP, RF, L-6, IL-10, TNF-α and INF-γ levels [42].
Tannic acid (TA)	(a) Natural TA’s molecular weight: 1701.20 g/mol [103]; low bioavailability due to its poor absorption and its low lipid solubility [104]. (b) It has anti-inflammatory effects by inhibiting paw edema through reduction of the activity of myeloperoxidase (MPO) enzyme in a formalin-induced paw edema model [105]. It possesses antidiabetic properties through inhibition of enzymes related to metabolism such as α-glucosidase and α-amylase [105]. It reduces the absorption of monosaccharides in the digestive tract and controls blood sugar levels [105]. It reduces aldose reductase and sorbitol dehydrogenase in the kidneys and attenuates diabetic kidney complications [105].	Phenylboronic acid (pPBA)-containing polymers [43]; poly(N-vinylpyrrolidone) (PVPON) [45].	Formation of phenylboronate ester bonds between polymeric phenylboronate and TA [43]. Formation of TA/poly(N-vinylpyrrolidone) (TA/PVPON) hydrogen-bonded multi-layers [45].	(a) PTNG (polymeric tannic acid-phenylboronic acid nanogel)’s size 250 nm [43]. (b) PTNG possesses anti-inflammatory effects decreasing PMA induced ROS production and TNF-α and IL-6 levels on murine macrophage (RAW264.7) cells [43]. PVPON/TA decreases immune cell infiltration and inflammatory chemokines and increases the anti-inflammatory M2 macrophages on autoimmune type 1 diabetes (T1D) mice model [45].
EGCG	(a) Natural EGCG’s molecular weight: 458.37 g/mol [106]. (b) It possesses antioxidant with anti-inflammatory properties and ROS, RNS scavenging characteristics [46].	Casein based biopolymer [47]. Chitosan based polymer [48].	Ultrasound-driven nanoencapsulation of (−)-Epigallocatechin Gallate–Glucosamine–Casein [47]. Ultrasound-driven nano-encapsulation of EGCG loaded chitosan—tripoly Phosphate (CS-TPP) NPs [48,49].	(a) Nano-EGCG-GA’s mean size: 186 nm [47]; high entrapment efficiency (up to 86.8%) [47]; high stability [47]; preservation of their physicochemical characteristics for a year after freeze-drying process [47]; 3-fold more time for casein degradation in gastric fluids [47]. (b) Nano-EGCG-GA shows 20.8% more profound inhibitory activity on human fibroblast-like synoviocytes-rheumatoid arthritis cells than the free EGCG-GA mixture [47]. Topical application of nano-EGCG reduces the levels of inflammatory cytokines and chemokines such as IL-1α, IL-1β, IL-4, IL-5, IL-6, IL-12, IL-13, and IFN-α and ameliorates the skin thickness, the erythema, the infiltration of inflammatory cells (mast cells, neutrophils, macrophages, and CD4^+^ T cells) and the angiogenesis in imiquimod induced murine psoriasic model [48].
Eugenol	Natural eugenol has pleiotropic activities, apart from the antioxidant [50].	Chitosan based polymer [50].	Nano-encapsulation of eugenol loaded chitosan–tripoly Phosphate (CS-TPP) NPs through centrifugation [50].	Nano-Eugenol significantly reduces the serum levels of malondialdehyde and Fork head Box O3 (FOXO3) protein and the expression of MCP1/CCL2 and TGF-β on the aggressive model of rheumatoid arthritis [50].
Lycopene (Lyc)	(a) Natural Lyc has low bioavailability due to its low absorption (10–30%) and its metabolic reactions, such as isomerization and oxidation that take place [62]. (b) It possesses anti-inflammatory properties through the inhibition of the NF-κΒ pathway [63].	Nanoemulsion droplets [63]; nanostructured lipid carriers (NLC) [64].	Lycopene-loaded emulsions [63]. High shear homogenization method [64].	(a) NF-Lyc’s mean size: 241 nm [63]; polydispersity index 0.284 [63]; ability to aggregate forming larger nanoparticles [63]; increased bioavailability compared to free Lyc [63]; low excretion rate by the liver and kidneys [63]. (b) NLCs shows increased uptake from psoriatic cells in psoriatic-like cellular model and preserves skin integrity in psoriatic skin [64].

## References

[1] Pisetsky D.S. (2023). Pathogenesis of autoimmune disease. Nat. Rev. Nephrol..

[2] Rui K., Peng N., Xiao F., Lu L. (2023). New insights into the functions of MDSCs in autoimmune pathogenesis. Cell Mol. Immunol..

[3] Schett G., Mackensen A., Mougialakos D. (2023). CAR T-cell therapy in autoimmune diseases. Lancet.

[4] Jung S.M., Kim W.-U. (2022). Targeted Immunotherapy for Autoimmune Disease. Immune Netw..

[5] Wojcik P., Gegotek A., Zarkovic N., Skrzydlewska E. (2021). Oxidative Stress and Lipid Mediators Modulate Immune Cell Functions in Autoimmune Diseases. Int. J. Mol. Sci..

[6] Papagiouvannis G., Theodosis-Nobelos P., Kourounakis P.N., Rekka E.A. (2021). Multi-target directed compounds with antioxidant and/or anti-inflammatory properties as potent agents for Alzheimer’s disease. Med. Chem..

[7] Papagiouvannis G., Theodosis-Nobelos P., Rekka E.A. (2025). A Review on Therapeutic Strategies against Parkinson’s Disease: Current Trends and Future Perspectives. Min. Rev. Med. Chem..

[8] Mannucci C., Casciaro M., Sorbara E.E., Calapai F., DiSalvo E., Pioggia G., Navarra M., Calapai G., Gangemi S. (2021). Nutraceuticals against Oxidative Stress in Autoimmune Disorders. Antioxidatns.

[9] Feczko T. (2021). Polymeric nanotherapeutics acting at special regions of body. J. Drug Deliv. Sci. Technol..

[10] Alshawwa S.Z., Kassem A.A., Farid R.M., Mostafa S.K., Labib G.S. (2022). Nanocarrier Drug delivery Systems: Characterization, Limitations, Future Perspectives and Implementation of Artificial Intelligence. Pharmaceutics.

[11] De R., Mahata M.K., Kim K.-T. (2022). Structure-Based Varieties of Polymeric Nanocarriers and Influences of Their Physicochemical Properties on Drug Delivery Profiles. Adv. Sci..

[12] Cappuccio de Castro K., Martins Costa J., Nogueira Campos M.G. (2020). Drug-loaded polymeric nanoparticles: A review. Int. J. Polym. Mater. Polym. Biomater..

[13] Su T., Feng X., Yang J., Xu W., Liu T., Zhang M., Ding J., Chen X. (2022). Polymer nanotherapeutics to correct autoimmunity. J. Control. Release.

[14] Zheng X., Sun K., Liu Y., Yin X., Zhu H., Yu H., Zhao W. (2023). Resveratrol-loaded macrophage exosomes alleviate multiple sclerosis through targeting microglia. J. Control. Release.

[15] Yavarpour-Bali H., Ghasemi-Kasman M., Pirzadeh M. (2019). Curcumin-loaded nanoparticles: A novel therapeutic strategy in treatment of central nervous system disorders. Int. J. Nanomed..

[16] Karaka E., Yarim M. (2023). Naringenin stimulates aromatase expression and alleviates the clinical and histopathological findings of experimental autoimmune encephalomyelitis in C57bl6 mice. Histochem. Cell. Biol..

[17] Nagasaki Y. (2018). Design and application of redox polymers for nanomedicine. Polym. J..

[18] Huang Y., Guo X., Wu Y., Chen X., Feng L., Xie N., Shen G. (2024). Nanotechnology’s frontier in combatting infectious and inflammatory diseases: Prevention and treatment. Signal Transduct. Target. Ther..

[19] Patel U., Rajasingh S., Samanta S., Cao T., Dawn B., Rajasingh J. (2017). Macrophage polarization in response to epigenetic modifiers during infection and inflammation. Drug Discov. Today.

[20] Gambini J., Inglés M., Olaso G., Lopez-Grueso R., Bonet-Costa V., Gimeno-Mallench L., Mas-Bargues C., Abdelaziz K.M., Gomez-Cabrera M.C., Vina J. (2015). Properties of Resveratrol: In Vitro and In Vivo Studies about Metabolism, Bioavailability, and Biological Effects in Animal Models and Humans. Oxid. Med. Cell. Longev..

[21] Singh C.K., George J., Ahmad N. (2013). Resveratrol-based combinatorial strategies for cancer management. Ann. N. Y. Acad. Sci..

[22] Park D., Jeong H., Lee M.N., Koh A., Kwon O., Yang Y.R., Noh J., Suh P.G., Park H., Ryu S.H. (2016). Resveratrol induces autophagy by directly inhibiting mTOR through ATP competition. Sci. Rep..

[23] Fei Q., Kent D., Botello-Smith W.M., Nur F., Nur S., Alsamarah A., Chatterjee P., Lambros M., Luo Y. (2018). Molecular Mechanism of Resveratrol’s Lipid Membrane Protection. Sci. Rep..

[24] Chen X., Zhu X., Ma L., Lin A., Gong Y., Yuan G., Liu J. (2019). A core-shell structure QRu-PLGA-RES-DS NP nanocomposite with photothermal response-induced M2 macrophage polarization for rheumatoid arthritis therapy. Nanoscale.

[25] Chountoulesi M., Demetzos C. (2020). Promising Nanotechnology Approaches in Treatment of Autoimmune Diseases of Central Nervous System. Brain Sci..

[26] Alizadeh A.M., Sadeghizadeh M., Najafi F., Ardestani S.K., Erfani-Moghadam V., Khaniki M., Rezaei A., Zamani M., Khodayari S., Khodayari H. (2015). Encapsulation of curcumin in diblock copolymer micelles for cancer therapy. BioMed Res. Int..

[27] Mohajeri M., Sadeghizadeh M., Najafi F., Javan M. (2015). Polymerized nano-curcumin attenuates neurological symptoms in EAE model of multiple sclerosis through down regulation of inflammatory and oxidative processes and enhancing neuroprotection and myelin repair. Neuropharmacology.

[28] Dolati S., Ahmadi M., Aghebti-Maleki L., Nikmaram A., Marofi F., Rikhtegar R., Ayromlou H., Yousefi M. (2018). Nanocurcumin is a potential novel therapy for multiple sclerosis by influencing inflammatory mediators. Pharmacol. Rep..

[29] Huang Y., Canup B.S.B., Gou S., Chen N., Dai F., Xiao B., Li C. (2021). Oral nanotherapeutics with enhanced mucus penetration and ROS-responsive drug release capacities for delivery of curcumin to colitis tissues. J. Mater. Chem. B.

[30] Qiao H., Fang D., Chen J., Sun Y., Kang C., Di L., Li J., Chen Z., Chen J., Gao Y. (2017). Orally delivered polycurcumin responsive to bacterial reduction for targeted therapy of inflammatory bowel disease. Drug Deliv..

[31] Mao K.L., Fan Z.L., Yuan J.D., Chen P.P., Yang J.J., Xu J., ZhuGe D.L., Jin B.H., Zhu Q.Y., Shen B.X. (2017). Skin-penetrating polymeric nanoparticles incorporated in silk fibroin hydrogel for topical delivery of curcumin to improve its therapeutic effect on psoriasis mouse model. Colloids Surf. B Biointerfaces.

[32] Kim D.K., Sim B.R., Khang G. (2016). Nature-Derived Aloe Vera Gel Blended Silk Fibroin Film Scaffolds for Cornea Endothelial Cell Regeneration and Transplantation. ACS Appl. Mater. Interfaces.

[33] Zhang Y., Xia Q., Li Y., He Z., Li Z., Guo T., Wu Z., Feng N. (2019). CD44 Assists the Topical Anti-Psoriatic Efficacy of Curcumin-Loaded Hyaluronan-Modified Ethosomes: A New Strategy for Clustering Drug in Inflammatory Skin. Theranostics.

[34] Bilia A.R., Bergonzi M.C., Isacchi B., Antiga E., Caproni M. (2018). Curcumin nanoparticles potentiate therapeutic effectiveness of acitrein in moderate-to-severe psoriasis patients and control serum cholesterol levels. J. Pharm. Pharmacol..

[35] Theodosis-Nobelos P., Rekka E.A. (2022). The Multiple Sclerosis Modulatory Potential of Natural Multi-Targeting Antioxidants. Molecules.

[36] Gokhale J.P., Mahajan H.S., Surana S.J. (2019). Quercetin loaded nanoemulsion-based gel for rheumatoid arthritis: In vivo and in vitro studies. Biomed. Pharmacother..

[37] Jeyadevi R., Sivasudha T., Rameshkumar A., Ananth D.A., Aseervatham G.S., Kumaresan K., Kumar L.D., Jagadeeswari S., Renganathan R. (2013). Enhancement of anti arthritic effect of quercetin using thioglycolic acid-capped cadmium telluride quantum dots as nanocarrier in adjuvant induced arthritic Wistar rats. Colloids Surf. B Biointerfaces.

[38] Souza K.S., Moreira L.S., Silva B.T., Oliveira B.P.M., Carvalho A.S., Silva P.S., Verri W.A., Sá-Nakanishi A.B., Bracht L., Zanoni J.N. (2021). Low dose of quercetin-loaded pectin/casein microparticles reduces the oxidative stress in arthritic rats. Life Sci..

[39] Halake K., Lee J. (2017). Functional hyaluronic acid conjugates based on natural polyphenols exhibit antioxidant, adhesive, gelation, and self-healing properties. J. Ind. Eng. Chem..

[40] Bhia M., Motallebi M., Abadi B., Zarepour A., Pereira-Silva M., Saremnejad F., Santos A.C., Zarrabi A., Melero A., Jafari S.M. (2021). Naringenin Nano-Delivery Systems and Their Therapeutic Applications. Pharmaceutics.

[41] Munir A., Faqir M., Yumna Z., Muhammad A.A., Mazhar I., Mubashar R., Muhammad U.M., Bushra A., Webster T.J., Ali S. (2021). Synthesis of naringenin loaded lipid based nanocarriers and their in-vivo therapeutic potential in a rheumatoid arthritis model. J. Drug Deliv. Sci. Technol..

[42] Mohanty S., Konkimalla V.B., Pal A., Sharma T., Si S.C. (2021). Naringin as Sustained Delivery Nanoparticles Ameliorates the Anti-inflammatory Activity in a Freund’s Complete Adjuvant-Induced Arthritis Model. ACS Omega.

[43] Yeo J., Lee J., Yoon S., Kim W.J. (2020). Tannic acid-based nanogel as an efficient anti-inflammatory agent. Biomater. Sci..

[44] Dosch M., Zindel J., Jebbawi F., Melin N., Sanchez-Taltavull D., Stroka D., Candinas D., Beldi G. (2019). Connexin-43-dependent ATP release mediates macrophage activation during sepsis. Elife.

[45] Barra J.M., Kozlovskaya V., Kharlampieva E., Tse H.M. (2020). Localized Immunosuppression With Tannic Acid Encapsulation Delays Islet Allograft and Autoimmune-Mediated Rejection. Diabetes.

[46] Tedeschi E., Menegazzi M., Yao Y., Suzuki H., Förstermann U., Kleinert H. (2004). Green tea inhibits human inducible nitric-oxide synthase expression by down-regulating signal transducer and activator of transcription-1alpha activation. Mol. Pharmacol..

[47] Zheng Y., Xiao L., Yu C., Jin P., Qin D., Xu Y., Yin J., Liu Z., Du Q. (2019). Enhanced Antiarthritic Efficacy by Nanoparticles of (-)-Epigallocatechin Gallate-Glucosamine-Casein. J. Agric. Food Chem..

[48] Chamcheu J.C., Siddiqui I.A., Adhami V.M., Esnault S., Bharali D.J., Babatunde A.S., Adame S., Massey R.J., Wood G.S., Longley B.J. (2018). Chitosan-based nanoformulated (-)-epigallocatechin-3-gallate (EGCG) modulates human keratinocyte-induced responses and alleviates imiquimod-induced murine psoriasiform dermatitis. Int. J. Nanomed..

[49] Khan N., Bharali D.J., Adhami V.M., Siddiqui I.A., Cui H., Shabana S.M., Mousa S.A., Mukhtar H. (2014). Oral administration of naturally occurring chitosan-based nanoformulated green tea polyphenol EGCG effectively inhibits prostate cancer cell growth in a xenograft model. Carcinogenesis.

[50] Jabbari N., Eftekhari Z., Roodbari N.H., Parivar K. (2020). Evaluation of Encapsulated Eugenol by Chitosan Nanoparticles on the aggressive model of rheumatoid arthritis. Int. Immunopharmacol..

[51] Chung C.H., Jung W., Keum H., Kim T.W., Jon S. (2020). Nanoparticles Derived from the Natural Antioxidant Rosmarinic Acid Ameliorate Acute Inflammatory Bowel Disease. ACS Nano.

[52] Asbaghi O., Ghanavati M., Ashtary-Larky D., Bagheri R., Rezaei K.M., Nazarian B., Nordvall M., Wong A., Dutheil F., Suzuki K. (2021). Effects of Folic Acid Supplementation on Oxidative Stress Markers: A Systematic Review and Meta-Analysis of Randomized Controlled Trials. Antioxidants.

[53] Ismail S., Eljazzar S., Ganji V. (2023). Intended and Unintended Benefits of Folic Acid Fortification—A Narrative Review. Foods.

[54] Sijilmassi O. (2019). Folic acid deficiency and vision: A review. Graefes Arch. Clin. Exp. Ophalmol..

[55] Theodosis-Nobelos P., Rekka E.A. (2024). The Antioxidant Potential of Vitamins and Their Implication in Metabolic Abnormalities. Nutrients.

[56] Nogueira E., Gomes A.C., Preto A., Cavaco-Paulo A. (2016). Folate-targeted nanoparticles for rheumatoid arthritis therapy. Nanomedicine.

[57] Srivastava S., Singh D., Singh M.R. (2018). Folate-Conjugated Superoxide Dismutase Adsorbed Over Antioxidant Mimicking Nanomatrix Frameworks for Treatment of Rheumatoid Arthritis. J. Pharm. Sci..

[58] Hu P., Tirelli N. (2012). Scavenging ROS: Superoxide dismutase/catalase mimetics by the use of an oxidation-sensitive nanocarrier/enzyme conjugate. Bioconjugate Chem..

[59] Periyathambi P., Sastry T.P., Anandasadagopan S.K., Manickavasagam K. (2017). Macrophages mediated diagnosis of rheumatoid arthritis using fibrin based magnetic nanoparticles as MRI contrast agents. Biochim. Biophys. Acta Gen. Subj..

[60] Schmidt G.P., Reiser M.F., Baur-Melnyk A. (2009). Whole-body MRI for the staging and follow-up of patients with metastasis. Eur. J. Radiol..

[61] Pan H., Chen L. (2021). Paramagnetic block copolymers: An effective tool for targeted radiography of rheumatoid arthritis. Mater. Express.

[62] Wang X.D. (2012). Lycopene metabolism and its biological significance. Am. J. Clin. Nutr..

[63] Moia V.M., Leal Portilho F., Almeida Pádua T., Barbosa Corrêa L., Ricci-Junior E., Cruz Rosas E., Magalhaes Rebelo Alencar L., Savio Mendes Sinfronio F., Sampson A., Hussain Iram S. (2020). Lycopene used as Anti-inflammatory Nanodrug for the Treatment of Rheumathoid Arthritis: Animal assay, Pharmacokinetics, ABC Transporter and Tissue Deposition. Colloids Surf. B Biointerfaces.

[64] Malgarim Cordenonsi L., Faccendini A., Catanzaro M., Bonferoni M.C., Rossi S., Malavasi L., Platcheck Raffin R., Scherman Schapoval E.E., Lanni C., Sandri G. (2019). The role of chitosan as coating material for nanostructured lipid carriers for skin delivery of fucoxanthin. Int. J. Pharm..

[65] Kumar S., Sharma S., Chattopadhyay S.K. (2013). The potential health benefit of polyisoprenylated benzophenones from Garcinia and related genera: Ethnobotanical and therapeutic importance. Fitoterapia.

[66] Miyashita K., Nishikawa S., Beppu F., Tsukui T., Abe M., Hosokawa M. (2011). The allenic carotenoid fucoxanthin, a novel marine nutraceutical from brown seaweeds. J. Sci. Food Agric..

[67] Dacaranhe C.D., Terao J. (2001). A unique antioxidant activity of phosphatidylserine on iron-induced lipid peroxidation of phospholipid bilayers. Lipids.

[68] Ma H.M., Wu Z., Nakanishi H. (2011). Phosphatidylserine-containing liposomes suppress inflammatory bone loss by ameliorating the cytokine imbalance provoked by infiltrated macrophages. Lab. Investig..

[69] Roberts R.A., Eitas T.K., Byrne J.D., Johnson B.M., Short P.J., McKinnon K.P., Reisdorf S., Luft J.C., DeSimone J.M., Ting J.P. (2015). Towards programming immune tolerance through geometric manipulation of phosphatidylserine. Biomaterials.

[70] Sheng Z., Ge S., Gao M., Jian R., Chen X., Xu X., Li D., Zhang K., Chen W.H. (2020). Synthesis and Biological Activity of Embelin and its Derivatives: An Overview. Mini Rev. Med. Chem..

[71] Cui P., Qu F., Sreeharsha N., Sharma S., Mishra A., Gubbiyappa S.K. (2020). Antiarthritic effect of chitosan nanoparticle loaded with embelin against adjuvant-induced arthritis in Wistar rats. IUBMB Life.

[72] Sedlak T.W., Saleh M., Higginson D.S., Paul B.D., Juluri K.R., Snyder S.H. (2009). Bilirubin and glutathione have complementary antioxidant and cytoprotective roles. Proc. Natl. Acad. Sci. USA.

[73] Lee Y., Kim H., Kang S., Lee J., Park J., Jon S. (2016). Bilirubin Nanoparticles as a Nanomedicine for Anti-inflammation Therapy. Angew. Chem. Int. Ed. Engl..

[74] Lee Y., Sugihara K., Gillilland M.G., Jon S., Kamada N., Moon J.J. (2020). Hyaluronic acid-bilirubin nanomedicine for targeted modulation of dysregulated intestinal barrier, microbiome and immune responses in colitis. Nat. Mater..

[75] Kim M.J., Lee Y., Jon S., Lee D.Y. (2017). PEGylated bilirubin nanoparticle as an anti-oxidative and anti-inflammatory demulcent in pancreatic islet xenotransplantation. Biomaterials.

[76] Zhu H.Q., Gao Y., Guo H.R., Kong Q.Z., Ma Y., Wang J.Z., Pan S.H., Jiang H.C., Dai W.J. (2011). Pretreatment with bilirubin protects islet against oxidative injury during isolation and purification. Transplant. Proc..

[77] Wang Q., Li W., Hu H., Lu X., Qin S. (2023). Monomeric compounds from traditional Chinese medicine: New hopes for drug discovery in pulmonary fibrosis. Biomed. Pharmacother..

[78] Ren S., Liu H., Wang X., Bi J., Lu S., Zhu C., Li H., Kong W., Chen R., Chen Z. (2021). Acupoint nanocomposite hydrogel for simulation of acupuncture and targeted delivery of triptolide against rheumatoid arthritis. J. Nanobiotechnol..

[79] Prasad S., Lillicrap D., Labelle A., Knappe S., Keller T., Burnett E., Powell S., Johnson K.W. (2008). Efficacy and safety of a new-class hemostatic drug candidate, AV513, in dogs with hemophilia A. Blood.

[80] Fitton J.H. (2011). Therapies from fucoidan; multifunctional marine polymers. Mar. Drugs.

[81] Cumashi A., Ushakova N.A., Preobrazhenskaya M.E., D’Incecco A., Piccoli A., Totani L., Tinari N., Morozevich G.E., Berman A.E., Bilan M.I. (2007). A comparative study of the anti-inflammatory, anticoagulant, antiangiogenic, and antiadhesive activities of nine different fucoidans from brown seaweeds. Glycobiology.

[82] Tanaka K., Ito M., Kodama M., Tomita M., Kimura S., Hoyano M., Mitsuma W., Hirono S., Hanawa H., Aizawa Y. (2011). Sulfated polysaccharide fucoidan ameliorates experimental autoimmune myocarditis in rats. J. Cardiovasc. Pharmacol. Ther..

[83] Tissot B., Daniel R. (2003). Biological properties of sulfated fucans: The potent inhibiting activity of algal fucoidan against the human compliment system. Glycobiology.

[84] Thring T.S., Hili P., Naughton D.P. (2009). Anti-collagenase, anti-elastase and anti-oxidant activities of extracts from 21 plants. BMC Complement. Altern. Med..

[85] Cui Y.Q., Zhang L.J., Zhang T., Luo D.Z., Jia Y.J., Guo Z.X., Zhang Q.B., Wang X., Wang X.M. (2010). Inhibitory effect of fucoidan on nitric oxide production in lipopolysaccharide-activated primary microglia. Clin. Exp. Pharmacol. Physiol..

[86] Zahariev N., Katsarov P., Lukova P., Pilicheva B. (2023). Novel Fucoidan Pharmaceutical Formulations and Their Potential Application in Oncology—A Review. Polymers.

[87] Baek Y., Jeong E.W., Lee H.G. (2023). Encapsulation of resveratrol within size-controlled nanoliposomes: Impact on solubility, stability, cellular permeability, and oral bioavailability. Colloids Surf. B Biointerfaces.

[88] Chung J.H., Lee J.-S., Lee H.G. (2020). Resveratrol-loaded chitosan–γ-poly (glutamic acid) nanoparticles: Optimization, solubility, UV stability, and cellular antioxidant activity. Colloids Surf. B Biointerfaces.

[89] Walle T. (2011). Bioavailability of resveratrol. Ann. N. Y. Acad. Sci..

[90] Sandhir R., Yadav A., Sunkaria A., Singhail N. (2015). Nano-antioxidants: An emerging strategy for intervention against neurodegenerative conditions. Neurochem. Int..

[91] Tanwal T., Saifullah S., Rehman J., Kawish M., Razzak A., Maharjan R., Imran M., Ali I., Roome T., Simjee S.U. (2021). Design of absorption enhancer containing self-nanoemulsifying drug delivery system (SNEDDS) for curcumin improved anti-cancer activity and oral bioavailability. J. Mol. Liq..

[92] Chen Y., Lu Y., Lee R.J., Xiang G. (2020). Nano Encapsulated Curcumin: And Its Potential for Biomedical Applications. Int. J. Nanomed..

[93] Lopresti A.L. (2018). The Problem of Curcumin and Its Bioavailability: Could Its Gastrointestinal Influence Contribute to Its Overall Health-Enhancing Effects?. Adv. Nutr..

[94] Fu Y.-S., Chen T.-H., Weng L., Huang L., Lai D., Weng C.-F. (2021). Pharmacological properties and underlying mechanisms of curcumin and prospects in medicinal potential. Biomed. Pharmacother..

[95] Gera M., Sharma N., Ghosh M., Huynh D.L., Lee S.J., Min T., Kwon T., Jeong D.K. (2017). Nanoformulations of curcumin: An emerging paradigm for improved remedial application. Oncotarget.

[96] Rawas-Qalaji M., Jagal J., Sadik S., Said Z., Ahmed I.S., Haider M., Hussain Z., Alhalaweh A. (2024). Assessment of enhancing curcumin’s solubility versus uptake on its anti-cancer efficacy. Colloids Surf. B Biointerfaces.

[97] Bertoncini-Silva C., Vlad A., Ricciarelli R., Fassini P.G., Miguel Suen V.M., Zingg J.-M. (2024). Enhancing the Bioavailability and Bioactivity of Curcumin for Disease Prevention and Treatment. Antioxidants.

[98] Hasnat H., Shompa S.A., Islam M., Alam S., Richi F.T., Emon N.U., Ashrafi S., Ahmed N.U., Chowdhury N.R., Fatema N. (2024). Flavonoids: A treasure house of prospective pharmacological potentials. Heliyon.

[99] Pangeni R., Kang S.-W., Oak M., Park E.Y., Park J.W. (2017). Oral delivery of quercetin in oil-in-water nanoemulsion: In vitro characterization and in vivo anti-obesity efficacy in mice. J. Funct. Foods.

[100] Cai X., Fang Z., Dou J., Yu A., Zhai G. (2013). Bioavailability of quercetin: Problems and promises. Curr. Med. Chem..

[101] De Gaetano F., Caridi F., Totaro N., Celesti C., Venuti V., Ginestra G., Nostro A., Tommasini S., Ventura C.A., Stancanelli R. (2025). Naringenin-Loaded Solid Lipid Nanoparticles: Physical-Chemical Characterization and In Vitro Antibacterial Activity. Pharmaceuticals.

[102] Duda-Madej A., Stecko J., Sobieraj J., Szymańska N., Kozłowska J. (2022). Naringenin and Its Derivatives-Health-Promoting Phytobiotic against Resistant Bacteria and Fungi in Humans. Antibiotics.

[103] Peşint G.B., Cemek K., Zenger O., Anar B.C., Başeğmez H.İ.O. (2023). Tannic acid purification from pomegranate peel via tannic acid imprinted particle-embedded cryogel column. J. Chromatogr. B.

[104] Ghasemian M., Kazeminava F., Naseri A., Mohebzadeh S., Abbaszadeh M., Kafil H.S., Ahmadian Z. (2023). Recent progress in tannic acid based approaches as a natural polyphenolic biomaterial for cancer therapy: A review. Biomed. Pharmacother..

[105] Jing W., Xiaolan C., Yu C., Feng Q., Haifeng Y. (2022). Pharmacological effects and mechanisms of tannic acid. Biomed. Pharmacother..

[106] Pathak N.M., Millar P.J.B., Pathak V., Flatt P.R., Gault V.A. (2018). Beneficial metabolic effects of dietary epigallocatechin gallate alone and in combination with exendin-4 in high fat diabetic mice. Mol. Cell. Endocrinol..

